# Emergency Transportation Interventions for Reducing Adverse Pregnancy Outcomes in Low- and Middle-Income Countries: A Systematic Review

**DOI:** 10.5334/aogh.2934

**Published:** 2020-11-18

**Authors:** Halimatou Alaofe, Breanne Lott, Linda Kimaru, Babasola Okusanya, Abidemi Okechukwu, Joy Chebet, Martin Meremikwu, John Ehiri

**Affiliations:** 1 University of Arizona, US; 2 University of Calabar, NG

## Abstract

**Objective:**

To assess the effect of emergency transportation interventions on the outcome of labor and delivery in low- and middle-income countries (LMICs).

**Methods:**

Eleven databases were searched through December 2019: Medline/PubMed, EMBASE, Web of Science, EBSCO (PsycINFO and CINAHL), SCIELO, LILACS, JSTOR, POPLINE, Google Scholar, the Cochrane Pregnancy and Childbirth Group’s Specialized Register, and the Cochrane Central Register of Controlled Trials. Methodological quality of included studies was assessed using the ROBINS-I tool.

**Results:**

Nine studies (three in Asia and six in Africa) were included: one cluster randomized controlled trial, three controlled before-and-after (CBA) studies, four uncontrolled before and after studies, and one case-control study. The means of emergency obstetric transportation evaluated by the studies included bicycle (n = 1) or motorcycle ambulances (n = 3), 4-wheel drive vehicles (n = 3), and formal motor-vehicle ambulances (n = 2). Transportation support was offered within multi-component interventions including financial incentives (n = 1), improved communication (n = 7), and community mobilization (n = 2). Two controlled before-and-after studies that implemented interventions including financial support, three-wheeled motorcycles, and use of mobile phones reported reduction of maternal mortality. One cluster-randomized study which involved community mobilization and strengthening of referral, and transportation, and one controlled before-and-after that implemented free-of-charge, 24-hour, 4 × 4 wheel ambulance and a mobile phone showed reductions in stillbirth, perinatal, and neonatal mortality. Six studies reported increases in facility delivery ranging from 12–50%, and one study showed a 19% reduction in home delivery. There was a significant increase of caesarian sections in two studies; use of motorcycle ambulances compared to car ambulance resulted in reduction in referral delay by 2 to 4.5 hours. Only three included studies had low risk of bias on all domains.

**Conclusion:**

Integrating emergency obstetric transportation with complimentary maternal health interventions may reduce adverse pregnancy outcomes and increase access to skilled obstetric services for women in LMICs. The strength of evidence is limited by the paucity of high-quality studies.

## Introduction

Pregnancy and childbirth are normal physiological processes. For most women in high-income countries, pregnancy is associated with a feeling of pride and immense joyous expectation [1]. However, for millions of women and their families in low- and middle-income countries (LMICs), where emergency obstetric care is limited, pregnancy and childbirth are a major cause of fear and anxiety [2]. While most women have normal pregnancies and safe deliveries, unanticipated obstetric complications and emergencies sometimes occur. Many causes of maternal mortality such as severe bleeding during and after childbirth, post-delivery infections, obstructed labor, and blood pressure disorders are preventable or treatable conditions [3]. In resource-poor settings, where many women deliver at home or in inadequately equipped health facilities, ensuring that those who develop obstetric emergencies during childbirth are quickly transported to facilities where they can receive quality emergency obstetric care can be the difference between life and death for the pregnant woman and her fetus. Unfortunately, referral to needed emergency obstetric care may not be possible for a plethora of reasons, including geography, cost, and lack of transportation [4].

Delays in reaching healthcare facilities for emergency obstetric care in LMICs can be reduced through implementation of transportation programs [5]. Transportation interventions for emergency obstetric care may include financing schemes that enable pregnant women to overcome barriers of transportation to health facilities for emergency obstetric care during labor and delivery. This may take the form of direct provision of transportation to healthcare facilities for pregnant women in need of emergency obstetric care. Examples include motorbike ambulances specially engineered for use in rough terrains in resource-limited communities, bicycle ambulances, cycle rickshaws, wheeled stretchers, canoes, and ox carts. [6,7,8,9]. Transportation interventions seek to decrease delay in reaching a health facility for emergency obstetric care, and they may contribute to reductions in adverse pregnancy and birth outcomes, including maternal deaths, stillbirths, and neonatal mortality in LMICs [10].

**Figure 1:**
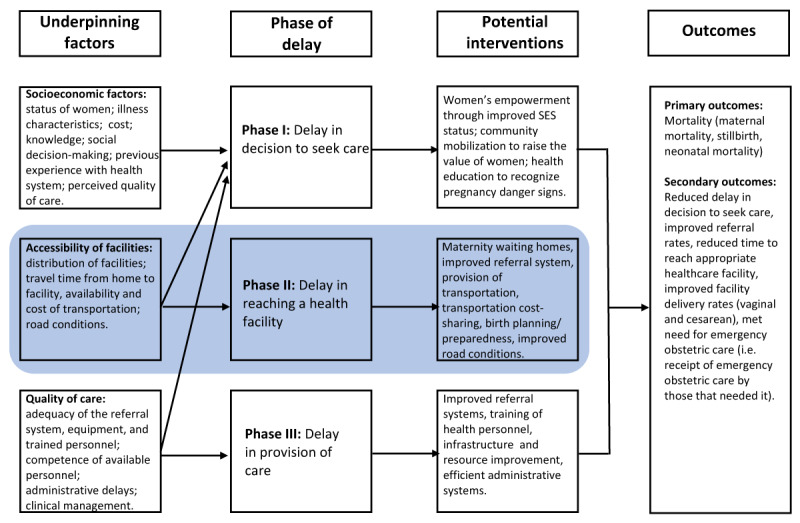
Conceptual framework for the review, based on the three-delay model.

In their seminal paper, “Too far to walk: Maternal mortality in context”, Thaddeus and Maine [11] presented a three-delay framework for analyzing maternal mortality in LMICs (Figure 1). Phase 1 delay refers to delay in the recognition of potentially life-threatening complications/emergencies and decision to seek care at a healthcare facility; Phase II refers to delay in time to reach a healthcare facility; Phase III delay refers to delays in receiving care once a woman reaches a healthcare facility [11,12,13,14]. Numerous social factors influence the decision to seek care, including lack of knowledge about the seriousness of complications, not knowing where to receive care, and/or waiting to receive permission from the husband or other family decision-makers [15]. Furthermore, an analysis of Demography and Health Survey (DHS) data from 41 countries showed that the most common obstacles to seeking obstetric care were financial barriers (>50%), challenges with transportation (37%), and distance (37%) [16]. Lack and high costs of transportation, poor road conditions, and time to arrange transport may also increase the time to reach a health facility [17,18,19]. Emergency obstetric transportation interventions are designed to address Phase II delays, i.e., delays that occur after the decision to seek care is made and before obtaining obstetric care. Thus, this review focused on assessment of the effects of emergency transportation interventions that were implemented to address Phase II delays aimed at reducing adverse pregnancy and birth outcomes in LMICs.

Faced with the challenge of unacceptably high maternal mortality rates, community organizations in many LMICs mobilize to provide free emergency obstetric transportation for pregnant women in need. For example, in several communities in northern Nigeria, where the maternal mortality ratio is more than twice the national average [20,21], the National Union of Road Transport Workers (NURTW) in conjunction with the Amalgamated Commercial Motorcycle Riders Association of Nigeria (ACOMORAN) operate jointly to provide emergency transportation for pregnant women. Other types of obstetric emergency transportation schemes include community health insurance and pre-payments, conditional cash transfers, vouchers, loans, and revolving funds aimed at alleviating the cost of transportation to needed emergency obstetric care [21,22,23,24,25,26]. In Kenya, the Maternal and Newborn Improvement (MANI) project uses a transport voucher to assist poor pregnant women to access health services [27]. Por et al [28]. established a voucher scheme and other financial incentives aimed at increasing access to skilled birth attendants for poor women in three rural health districts in Cambodia. Evaluation of this program showed that the scheme increased facility-based deliveries from 16.3% to 44.9% over a two-year period. This marked increase in skilled birth attendance was attributed to lessened financial burden on families [28,29]. Similar maternal voucher schemes aimed at reducing transport barriers for poor pregnant women have been described in Bangladesh [5] and Pakistan [30].

Although many obstetric emergency transportation interventions are being implemented in LMICs, there is limited empirical evidence to show their effect on reducing adverse outcomes associated with labor and delivery. The only available study on the subject is a 2015 systematic review that focused on only community-based loan funds for transportation during obstetric emergencies in developing countries [31]. This review demonstrated that compared to women in the control communities, those in sites where community-based loan funds were implemented experienced less maternal mortality, a higher rate of facility-based deliveries, and increased utilization of emergency obstetric care. This review is limited in scope, however, given that loan funds represent only one type of intervention that can be implemented to reduce the financial barriers that women in LMICs face in accessing transportation for obstetric care. To increase the scope of available evidence, this review summarized and critically appraised available data on the effect of all forms of interventions and financing mechanisms to promote transportation for emergency obstetric care in LMICs. Findings from this review may help to inform the global debate on access to routine and emergency obstetric services in LMICs.

## Methods

The protocol for this review was registered in PROSPERO (ID: CRD42017080092), and the review followed standard systematic review methods [16] and the Preferred Reporting Items for Systematic Reviews and Meta-analyses (PRISMA) [32] [Supplement 1]. This study did not require approval from the Internal Review Board because it used data from published studies.

## Search Strategy

We searched the following databases through December 30, 2019: Medline/PubMed, EMBASE, Web of Science, EBSCO (PsycINFO and CINAHL), SCIELO, LILACS, JSTOR, POPLINE, Google scholar, the Cochrane Pregnancy and Childbirth Group’s Specialized Register, and the Cochrane Central Register of Controlled Trials. No date or language restrictions were applied. The search strategy is described in detail in a previously published review protocol [33].

## Inclusion Criteria

**Figure 2:**
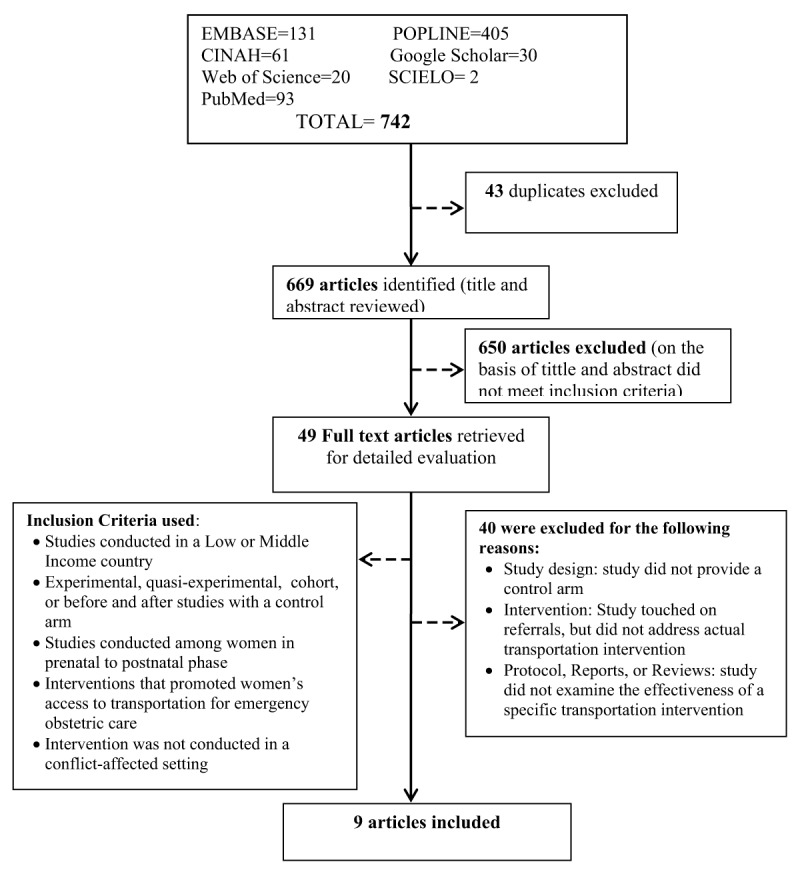
Literature Search Process and Results.

We included quasi-experimental studies, randomized controlled trials, controlled before-and-after studies, and cohort studies with control that assessed the effect of transportation interventions on pregnancy outcomes in LMICs. The target population were women who had prenatal, intrapartum, or post-natal care for an obstetric complication and were referred from the community or a primary health care center to a higher-level facility that could provide emergency obstetric care. The interventions of interest in this review included direct provision of transportation services as well as financing schemes or in-kind initiatives that enabled financial challenged pregnant women to overcome barriers of transportation to health facilities for emergency obstetric care in the prenatal period, during labor, delivery, or up to 42 days after delivery (postpartum period). Detailed description of the interventions of interest and conceptual framework is described in this review’s published protocol [33] and summarized in Figure 2. Comparison groups were women who had no transportation interventions for prenatal, intrapartum, or post-natal obstetric complications. Primary outcomes included mortality (stillbirth, maternal mortality, and neonatal mortality). Secondary outcomes included reduced delay in access to care, facility referral rates, time taken to reach appropriate healthcare facility, facility delivery rates, and met need for emergency obstetric care (i.e., receipt of emergency obstetric care by those that needed it). This review was restricted to studies conducted in countries designated as LMIC according to the World Bank’s classification [34].

## Study Selection

A study eligibility form was used to screen studies for inclusion [33]. Two reviewers independently screened the titles and abstracts of citations to assess their eligibility for inclusion. Thereafter, the full texts of eligible studies were independently reviewed by two review authors. Disagreements were resolved by discussions within the review team.

## Data Extraction

Using a modified Cochrane Collaboration’s data extraction form, two reviewers independently extracted data from eligible studies [35]. Data were extracted on study setting, design, participants’ characteristics, interventions, controls, and duration of follow-up. Study sample size, age, and data collection methods were recorded (Tables 1, 2, 3). Where necessary, authors of included studies were contacted for additional information or missing data.

**Table 1:** Characteristics of included studies.

Author (year)	Objectives	Study Design	Study Population	Intervention and Follow-up	Outcomes Measured	Key Results	Critical Appraisal

Lungu et al. (2000)	To evaluate the effectiveness of two interventions (bicycle ambulances and established transport plans) in decreasing home delivery rates	Case-control study	Women of childbearing age who delivered in Nsanje District of Malawi	Two villages provided with bicycle ambulances and two developed community transport plans Control group (no intervention) Follow-up period = 6 months	Home deliveries	Bicycles ambulances: 51.2% Transport plan: 9.8% Home deliveries in case villages decreased from 37% to 18%	No pre- and post-home deliveries data for control group
					Referrals	Control: 39% Bicycle ambulances: 20% of referrals to the health facilities were for obstetric reasons.	No pre- and post-home referrals data for all three groups No during intervention data of referrals for transport plan intervention and control group
					Transport time	Approximately 90 minutes required for travel with both interventions. No significant difference between all three groups	No pre- and post-transport time results for all three groups
					Cost-effectiveness	Bicycles ambulances: MK15 Transport plan: MK 0.30 Control: MK 0	No pre- and post-cost-effectiveness results for all three groups
De Costa et al. (2009)	To evaluate the effectiveness of financial support for transportation in reducing maternal deaths	Control before-after study	Women 15–45 years of age from scheduled castes and tribes as well as those who live below the poverty line in central India	Financial support for referrals needed by pregnant mothers and incentives for early registration of pregnancy Training of all health care paramedical staff and traditional birth attendants Control group (no intervention Follow-up period = 12 months	Maternal death	Intervention: pre (27); post (12) Control: intervention year (46)	No pre- and post-maternal deaths, live births, maternal mortality rations, and maternal death occurring at home
					Live births	Intervention: pre (5,084); post (5,221) Control: intervention year (7,662)	
					Maternal mortality ratios	Intervention: pre (531); post (249) Control: intervention year (600)	
					Maternal death occurring at home	Intervention: pre (55.6%); post (25%) Control: intervention year (58.7%) 89% of deliveries occurred at home in intervention block	
					Post-partum death	Intervention: pre (55.6%); post (25%) Control: intervention year (58.7)	No pre- and post- post-partum death data for control group
					Referral support	Intervention: 23.8% advised referral availed the referral benefits.	No pre- and post-referral data for both groups No intervention year data of referrals for control group
Mucunguzi et al. (2014)	To evaluate the effectiveness of a free-of-charge 24-hour ambulance and communication services intervention on emergency obstetric care outcomes	Control before-after study	Pregnant women from two districts of Northern Uganda	A 4 × 4 wheel ambulance available 24-hours and 7 days a week. Mobile phone and airtime to communicate with the ambulance team and the referral facility Control group (no intervention) Follow-up period = 36 months	Hospital stillbirths per 1000 births	Intervention: pre (46.6%); post (37.5%)	No pre- and post-stillbirth’s data for control group
					Hospital deliveries	Intervention: pre (1090); post (1646) Control: pre (1776); post (1810) Hospital deliveries increased by over 50% in intervention district	
					Caesarean sections rates	Intervention: pre (0.57%); post (1.21%) Control: pre (0.51%); post (0.58%) No significant increase in the control district	
					Cost of intervention	USD 1,875 per month.	
Prinja et al. (2014)	To assess the extent and pattern of NAS utilization, and whether NAS service has improved the utilization of public sector facilities for institutional deliveries	quasi-experimental design uncontrolled before-and-after	Pregnant women from Ambala, Hisar, and Narnual districts in Haryana state, India	*Haryana Swasthya Vaahan Sewa* (HSVS), now known as National Ambulance Service (NAS) – a government managed referral transport system with its administration decentralized to district level	Institutional deliveries	Ambala (OR = 137, 95% CI = 22.4–252.4); Hisar (OR = 215, 95% CI = 88.5–341.3) districts; Narnaul (OR = 4.5, 95% CI = –137.4 to 146.4) Institutional deliveries in Haryana rose significantly after the introduction of HSVS service, however, no significant increase was observed in Narnaul district.	No pre- and post-institutional delivery actual numbers; just an interrupted time series analysis.
Goudar et al. (2015)	To assess whether community mobilization and interventions to improve emergency obstetric and newborn care reduced perinatal and neonatal mortality rates	Cluster-randomized controlled trial	Pregnant women from 20 geogra-phically defined clusters in Belgaum, India	The intervention engaged and mobilized community, strengthened community-based stabilization, referral, and transportation, and improved quality of care at facilities in 10 clusters. Control group (no intervention) Follow-up period = 24 months	Neonatal mortality rate	Intervention: pre (26.7); post (18.4) Control: pre (21.2); post (24.1)	
					Perinatal mortality rate	Intervention: pre (52.7); post (37.8) Control: pre (47.9); post (44.2) No statistical significance was reached for both mortality outcomes.	
					Transportation	Intervention: pre (74.9%); post (87.1%) Control: pre (77.9%); post (98%)	
					Caesarean section	Intervention: pre (8.6%); post (13.1%) Control: pre (8.2%); post (13.4%) No significant difference between both groups	
					Facility birth rates	Intervention pre (87%); post (94%) Control pre (85%); post (93%)	
Patel et al. (2016)	To evaluate the impact of community-engaged emergency referral system in improving survival in impoverished rural Ghanaian communities	Control before-after study	Individuals living in the Upper East Region in Ghana	A fleet of 3-wheeled motorcycles known as Motorkings served as emergency transport vehicles Dual-SIM mobile phones distributed to health facilities, health workers, and volunteer drivers Control group (no intervention) Follow-up period = 24 months	Maternal mortality ratio	Intervention: pre (618); post (201) Control: pre (326); post (261)	No pre- and post-data on referrals and deliveries as well as caesarian delivery rates data for both groups. Only differences-in differences estimates were provided.
					Referrals into district hospitals from health centers	Intervention: Increase referrals into district hospitals from health centers by > 12 patients per month (P < 0.005)	
					Hospital deliveries	Intervention: No significant effect on the number of hospital deliveries (P > 0.05)	
					Cesarean delivery rate	Intervention: No significant effect on the cesarean delivery rate (P > 0.05)	
Fournier et al. (2009)	To evaluate the effect of a national referral system that aims to reduce maternal mortality rates through improving access to and the quality of emergency obstetric care in rural Mali (sub-Saharan Africa)	Quasi-experimental uncontrolled before-and-after	Women with obstetric complications who are referred by community health centres and have benefited from all components of the system, and women who are self-referred to the district health centre.	**Intervention:** The maternity referral system aimed to: Improve communication and transport opportunities to eliminate delays in the delivery of emergency obstetric services Alternative funding options, including community cost-sharing schemes, are accessed to eliminate financial barriers to obstetric care Training and equipment provided to improve the clinical management of obstetric emergencies **Follow-up:** The effect was evaluated in these time periods: P-1: year before the intervention; P0: year of the intervention; P1: 1 year after the intervention P2: 2 years after the intervention	Institutional deliveries	Institutional deliveries over expected deliveries: P-1: 9871/52045 (19%) P0: 15576/58453 (27%) P1: 16573/51868 (32%) P2: 19235/48846 (39%)	
					Obstetric emergencies treated	Referred Obstetric Emergencies treated over all obstetric emergencies: P-1: 143/475 (30%) P0: 273/658 (41%) P1: 246/571 (43%) P2: 452/913 (50%)	
Hoffman et al. (2008)	To assess whether motorcycle ambulances are more effective method of reducing referral delay for obstetric emergencies than a car ambulance, and to compare investment and operating costs with those of a 4-wheel drive car ambulance	Uncontrolled before-and-after	Women with obstetric complications in Mangochi district, Malawi	**Intervention:** Three motorcycle ambulances, consisting of a 250 cc Yamaha motorcycle with sidecar, which could carry 2 adults, were stationed at three remote rural health centers (Makanjira, Mase, and Phirilongwe) in Mangochi district, Malawi. **Follow-up:** Intervention occurred over a 12-month period from October 2001 to September 2002.	Reduction of 2^nd^ delay	Median referral delay was reduced by 2–4.5 hours (35%–76%).	No pre- or post- data on facility deliveries before or after intervention as a result in reduction of 2^nd^ delay
					Cost-effectiveness	Purchase price of a motorcycle ambulance was 19 times cheaper than for a car ambulance. Annual operating costs of a motorcycle ambulance were US $508, which was almost 24 times cheaper than for a car ambulance.	
Ngoma et al. (2019)	Addresses how Saving Mothers Giving Life (SMGL) Initiative in Uganda and Zambia implemented strategies specifically targeting the second delay, including decreasing the distance to facilities capable of managing emergency obstetric and newborn complications, ensuring sufficient numbers of skilled birth attendants, and addressing transportation challenges	Uncontrolled before-and-after	Pre-natal women in SMGL districts in Uganda and Zambia	**Intervention:** A key element of the SMGL initiative was the creation of an integrated communication and transportation system that functions 24 hours a day, 7 days a week, to encourage and enable pregnant women to access delivery care facilities. Both Uganda and Zambia led several efforts to facilitate transportation to and between facilities. **Follow-up:** The SMGL initiative in both Uganda and Zambia operated within 3 phases: Phase 0: Design and start-up (June 2011 to May 2012) Phase 1: Proof of concept (June 2012 to December 2013) Phase 2: scale-up and scale-out (January 2014 to October 2017). During Phase 2, SMGL expanded its presence in Uganda from 4 districts to 13 districts, and in Zambia from 6 to 18 districts.	Facility deliveries	Uganda observed a +45% and Zambia +12% relative change in deliveries in Emergency Obstetric and Newborn Care (EmONC) facilities between Jun 2012 and Dec 2016. Uganda observed a +200% and Zambia +167% relative change in the number of basic EmONC facilities Jun 2012 and Dec 2016. Uganda observed a +143% and Zambia +25% relative change in the number of comprehensive EmONC facilities Jun 2012 and Dec 2016. Zambia observed a +31% and Uganda -3% relative change in health facilities that reported having available transportation (motor vehicle or motorcycle). However, Uganda had a different transport intervention Institutional delivery supported by Baylor transportation vouchers that observed a +258% increase Jun 2012 and Dec 2016.	

**Table 2:** Intervention components implemented by included studies to improve transportation and reduce delay for obstetric emergencies.

Study	Intervention Components				Description of Intervention Components

	Transportation	Communication	Cost-Sharing	Community Mobilization	

De Costa	No	No	Yes	Yes	Financial support was provided for transportation of emergency referral cases and any accompanying health worker. Incentives also existed for early registration of pregnancy, receipt of antenatal care, and detection of high-risk pregnancies. Transportation (tractors, vans, other modes of transport) was arranged through informal contacts (mobilized community).
Fournier	Yes	Yes	Yes	No	A non-descript ambulance service was improved through intervention between health facilities only. Communication was improved with radios. Costs for transportation were shared by local government, local health services, community health associations, and a co-pay from the pregnant women.
Goudar	No	Yes	Yes	Yes	Community-based workers were trained to effectively communicate with transportation facilitators and hospital staff. Emergency funds were created using personal savings or local resources. Community Action Cycle was used to empower communities to identify, prioritize, and act on maternal and neonatal health problems. This included establishing birth plans and arranging alternative emergency local transportation.
Hofman	Yes	No	Yes	No	Three motorcycle ambulances with sidecars were stationed at remote rural health centers. The ambulances were operated by trained Health Surveillance Assistants. They picked women up from their homes and transported them between health facilities (only transportation between health facilities was evaluated in this study). Transportation was provided free-of-charge.
Lungu	Yes	No	Yes	No	Two communities used bicycle ambulances and two communities developed transport plans. Communities fundraised to create a maintenance reserve, as determined by financial committees in each site. Communities with transport plans implemented a MK 10 flat rate charge for each trip to the health center.
Ngoma	Yes	Yes	Yes	Yes	Various ambulances were procured for different study communities: 4 × 4 ambulances (Uganda and Zambia), motorized tricycle ambulances (Uganda), bicycle ambulances (Zambia), and motorcycle ambulances (Zambia). Transportation was available 24/7, for transport to facilities and referral between facilities. District transportation committees were established or strengthened to coordinate ambulances (Uganda and Zambia). Two-way radios (Zambia) and cell phones and airtime (Zambia) were supplied to facilitate communication. Transportation vouchers and village-level savings programs were used to alleviate cost barriers (Zambia). Village health teams and action groups were trained to encourage birth preparedness and to escort women to facility.
Mucunguzi	Yes	Yes	Yes	No	One 4 × 4 ambulance was stationed at the district hospital and provided transportation free-of-charge, 24/7, between health facilities only. Mobile phones and airtime were provided to each health facility to facilitate communication.
Patel	Yes	Yes	Yes	Yes	24 three-wheeled motorcycles with structural modifications for patient safety and comfort were stationed at health centers, health posts, and at homes of chiefs or assembly men in communities with no health facilities. They transported all pregnant women (emergency and normal cases) free of charge. Dual-SIM mobile phones and airtime were distributed to health facilities, health workers, and drivers. A phone line dedicated to receiving incoming calls was established at the tertiary referral point in each ward. Community meetings were held to distribute emergency phone numbers, share information about the ambulance service, and distribute posters to be hung at health facilities and community gathering places.
Prinja	Yes	Yes	Yes	No	240 traditional ambulances were stationed at community health centers and primary health centers. Transport was free for pregnant women, neonates, and postnatal cases. A 24/7 call center, with a toll-free emergency number, dispatched ambulances using GIS.

**Table 3:** Description of ambulance vehicles used by included studies, with pros and cons for each type of transportation.

Ambulance Type	Description of Vehicle	Pros for Mode of Transport	Cons for Mode of Transport

Formal ambulance [40] 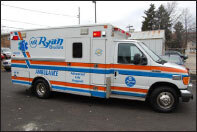	A large vehicle, such as a van, with four wheels that transports patients in a rear compartment, usually while laying down. May be stocked with life-saving equipment and medications. Usually equipped with sirens and insignia so that the vehicle is easily identified.	Can accommodate multiple individuals, such as a patient and their family/caregivers. Patients can receive basic medical attention prior to arrival at health facility. May utilize GIS to reach patients quickly.	Cannot reach patients in areas with rough terrain. Expensive. Requires a professional driver.
4 × 4 Landcruiser ambulance [44,45] 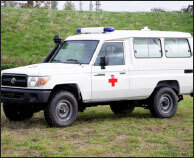	A high-clearance vehicle with four-wheel drive that transports patients in a rear compartment, either laying or sitting.	Can accommodate multiple individuals. May pick up health workers for emergencies at night. Can handle more rugged terrain than a traditional ambulance.	Still not able to access narrow roads or routes with very poor road conditions. May be inoperable during rainy season or inclement weather. Expensive. Requires a professional driver. May be misused for non-health-related activities.
Motorcycle or motorized tricycle ambulance [38,42,45] 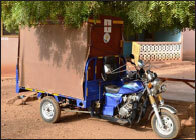	A motorcycle may be fitted with an open or closed sidecar, carriage, or wheeled stretcher that carries a patient and up to one other person.	Can handle more rugged terrain than all other types of transportation. Able to navigate narrow passages with poor road conditions. Can therefore, operate year-round, even during rainy season or inclement weather. Less expensive than other motorized vehicles. Can be operated by trained volunteers or community health workers.	Has limited capacity to carry multiple individuals. Mixed reviews from patients about comfort. Leaves the driver exposed to the elements/vulnerable to weather. May not be preferred by drivers for use at night, due to safety concerns.
Bicycle ambulance [41,45] 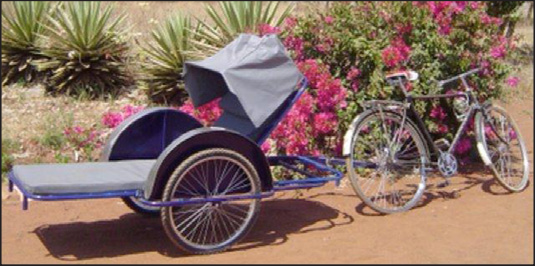	A bicycle may be fitted with an open or enclosed trailer, carriage, or wheeled stretcher that carries one patient only.	Inexpensive. Maintenance can be performed easily. Can be operated by a wide range of people.	Can usually only carry the patient. May not be culturally acceptable. May not be comfortable. May not offer as much privacy to patients as other forms of transportation. May not reduce time to health facility.

## Quality Assessment

Two review authors assessed the quality of included studies using the Risk Of Bias In Non-randomized Studies of Interventions (ROBINS-I) tool [36]. The domains of the tool include confounding, participant selection, intervention classification, deviation from intended intervention, missing data, measurement of outcomes, and selection of results reported. Disagreements between the two assessors were resolved by discussion and consensus, with arbitration by a third reviewer as required. In line with ROBINS-I guidelines [36], each criterion was scored as “low risk,” “moderate risk,” “serious risk,” “critical risk,” or “no information” (Table 4). An overall risk of bias judgment was made in accordance with the guidelines of ROBINS-I [36].

**Table 4:** Methodological quality assessment of included studies using the ROBIS-I tool.

Author	Confounding	Selection of Participants	Classification of Intervention	Intervention Deviation	Missing Data	Measurement of outcomes	Selection of result Reported	Overall

**De Costa et al. 2009**	Moderate	Low	Moderate	Low	Serious	Low	Low	Serious
**Fournier et al. 2009**	Moderate	Moderate	Low	No information	Low	Serious	Low	Serious
**Goudar et al. 2015**	Low	Low	Low	Low	Low	Low	Low	Low
**Hofman et al. 2008**	Serious	Low	Low	Low	Serious	Moderate	Low	Serious
**Lungu et al. 2000**	Moderate	Low	Low	Low	Low	Low	Low	Moderate
**Mucunguzi et al. 2014**	Critical	Low	Low	Low	Moderate	Moderate	Low	Critical
**Ngoma et al. 2019**	Critical	Low	Low	Low	Low	Low	Serious	Critical
**Patel et al. 2016**	Low	Low	Low	Low	Low	Low	Low	Low
**Prinja et al. 2014**	Low	Low	Low	Low	Low	Low	Low	Low

## Data Analysis

There was significant clinical heterogeneity of included studies, which made it impossible to perform a meta-analysis. Therefore, a systematic review of the nine eligible studies was conducted by summarizing, comparing, and contrasting the extracted data. We did not do a GRADE evidence summary of the systematic review because some of the included studies have multiple intervention sub-components, which might make a summary of evidence misleading. For instance, regarding maternal mortality, a primary outcome, an included study (De Costa et al. [37]) provided financial support, while another study (Patel et al. [38]) used a combination of three-wheeled motorcycles and dual-SIM phones to achieve reduced maternal mortality.

## Results

The search identified 742 titles, whose screening, along with their abstracts, resulted in 49 potentially eligible studies. Following full text review, 40 were excluded for not meeting the inclusion criteria, resulting in the nine that were included in this review (Figure 2) [37,38,39,40,41,42,43,44,45].

## Sample Characteristics

As shown in Table 1, the included studies, all published in English between 2000 and 2019, were conducted in Asia, (India [37,39,40]) and Africa (Malawi [41,42], Mali [43], Uganda [44,45], Zambia [45], and Ghana [38]).

Three controlled before-and-after studies [37,38,44], four uncontrolled before and after studies [40,42,43,45], one cluster randomized controlled trial [39], and one case-control study [41] were included. Eight of the nine studies were conducted in rural settings. Participants were identified through obstetric cases and hospital records. There was significant clinical heterogeneity in sample characteristics, including size (157 to 6926 pregnant women), participants’ age (15–45 years), exclusion criteria, and a marked variation in length of follow-up (6–36 months).

## Intervention Characteristics

Interventions used various strategies: (a) transportation financing strategies, (b) communication and transport systems, and (c) community mobilization (Table 2). Six studies (66.7%) provided a new form of transportation to communities including four-wheel drive, motorcycle, or bicycle ambulances [38,40,42,44], while one improved an already existing ambulance service [43]. The remaining two studies mobilized communities to arrange local means of transportation [37,39]. Communication was enhanced by providing radios and mobile cell phones to health workers and drivers in six studies [38,39,40,43]. All included studies attempted to address cost of transportation as a barrier by offering free transportation services (n = 5) [30,38,42,44], transportation vouchers (n = 1) [45], or using a cost-sharing strategy such as community emergency funds or savings groups (n = 5) [37,39,41,43,45].

## Effects of Intervention

### Primary Outcomes

Among the nine included studies, two reported data on maternal mortality [37,38], one on stillbirth [44], and one on neonatal and perinatal mortality [39].

#### Maternal mortality

After adjusting for number of women, De Costa et al. [37] (risk of bias: critical) reported reduced maternal death in Uganda in the financial support group during the project year relative to the previous year (0.16% vs. 24.5%) and compared to the control group (0.16% vs. 22.5%) in the same year, respectively. Patel et al. [38] reported the use of three-wheeled motorcycles and dual-SIM phones reduced maternal mortality ratio by 417 in the intervention group, compared with a decrease of 65 in the control group over the 24-month study (risk of bias: low).

#### Neonatal mortality and stillbirth

The two studies that reported on child mortality showed reductions in stillbirth, neonatal, and perinatal mortality [39,44]. Mucunguzi et al. [44] (risk of bias: critical) demonstrated that free-of-charge, 24-hour, 4 × 4 wheel ambulance and a mobile phone decreased hospital stillbirths per 1000 births by 9.1 in the intervention group. A cluster-randomized study in India that involved community mobilization and strengthening of referral and transportation reported reductions in perinatal mortality by 14.9 and neonatal mortality by 8.3 in the intervention group, compared to a decrease of 3.7 and an increase of 8.3 in the control group over the 6-month study, respectively (Goudar et al., [39] risk of bias: low).

### Secondary Outcomes

#### Facility or home deliveries

Seven included studies reported data on facility or home deliveries [38,39,41,43]. One study (Mucugunzi et al., [44] risk of bias: critical) reported a 50% increase in facility delivery, while Goudar et al. [39] (risk of bias: low) demonstrated a 7-point increase in facility deliveries. Fournier et al. [43] (risk of bias: serious) reported a higher proportion of institutional deliveries of 32% one-year post-intervention, compared to 19% pre-intervention. In the study by Ngoma et al. [45] (risk of bias: critical), facility deliveries increased by 21% and 27%, four years after the start of intervention in Uganda and Zambia, respectively. Prinja et al. [40] (risk of bias: low) found institutional deliveries rose significantly after the introduction of National Ambulance Service (NAS) in high NAS utilization (OR = 137, 95% CI = 22.4–252.4) and medium NAS utilization (OR = 215, 95% CI = 88.5–341.3) districts. However, no significant increase was observed in low NAS utilization district (OR = 4.5, 95% CI = –137.4–146.4). Similarly, Patel et al. [38] (risk of bias: low) reported no significant effect of community-engaged emergency referral system on hospital deliveries in Ghana. Finally, Lungu et al. [41] (risk of bias: moderate) reported a 19% reduction in home deliveries by pregnant women in the intervention group.

#### Caesarian sections

Three included studies [38,39,44] reported cesarean section outcomes. In Uganda (risk of bias: critical) [44] and India (risk of bias: low), [39] the average cesarean sections rates in the intervention district increased by 0.64% and 4.5%, respectively. However, no significant effect was observed by Patel et al. [38] (risk of bias: low).

#### Referral services

Two included studies reported referral services [37,41]. In the Lungu et al. [41] (risk of bias: moderate) study, where bicycle ambulances or community transport plans were provided in intervention villages, 20% of referrals were for obstetric reasons, while others reason for referrals were general medical cases. De Costa et al. [37] (risk of bias: serious) reported only 23.8% of the pregnant women referred used the referral services despite financial support for referrals and incentives for early registration of pregnancy.

#### Time of transport

Three included studies reported time of transport [40,41,42]. Lungu et al. [41] (risk of bias: moderate) found no significant difference in time to health facility between women using a bicycle ambulance, a community transport plan, or neither. Hoffman et al. [42] (risk of bias: serious) reported a decrease in referral delay by 2 to 4.5 hours following use of motorcycle ambulances in three remote health centers in Malawi. In India, Prinja et al. [40] (risk of bias: low) reported an average time taken by the national ambulance to reach the emergency site and to transport the patient to a health facility, of 17.5 minutes and 48 minutes, respectively.

#### Cost

Two included studies reported on cost of various transportation interventions. Lungu et al. [41] (risk of bias: moderate) reported the cost of bicycle ambulances at MK15 (0.02 USD) and community transport plan at MK0.30 (0.0004 USD) per institutional delivery. Fournier et al. [43] (risk of bias: serious) observed that purchasing a motorcycle ambulance was 19 times cheaper and the operational cost was 24 times cheaper than using a car ambulance. Characteristics of different types of ambulances used by studies in this review, such as the ability to reduce travel time or cost-effectiveness, are presented in Table 3.

### Quality Assessment

Table 4 summarizes the methodological quality of studies included in this review. Three included studies were judged as having low risk of bias on all domains [38,39,40]. One study was judged as having moderate risk of bias due to confounding [41]. Three included studies were judged as having serious risk of bias due to missing data and measurement of outcomes [37,42,43]. Two included studies were judged as having critical risk of bias due to confounding [38,45].

## Discussion

This systematic review found limited but promising evidence that emergency transportation interventions in LMICs may be effective in: i) reducing maternal and child mortality, ii) increasing health facility delivery significantly, iii) increasing caesarian sections for women in need of such service, and iv) reducing referral delay. Evidence suggests that integrating emergency obstetric transportation with complementary maternal health interventions (e.g., improved communication) reduces adverse pregnancy and childbirth outcomes. Two of the nine included studies reported maternal mortality reduction, and two showed reductions in stillbirths as well as neonatal and perinatal mortality. Six studies reported increases in facility deliveries, ranging from 12–50%; one study demonstrated 19% reduction in home delivery. There was a significant increase in caesarian sections in two studies, while 23.8% of women referred eventually used the service. There was also a reduction in referral delay by 2 to 4.5 hours with the use of motorcycle ambulances compared to car ambulance. However, the type, quality, and scope of transportation interventions varied significantly, and a critical mass of complementary activities is needed to achieve maximum impact.

Different emergency transport services were used, including bicycle ambulances, motorcycle or motorized tricycle ambulances, 4-wheel drive vehicles, and formal ambulances, each with its own advantages and disadvantages. Formal ambulances and 4 × 4 vehicles, while able to accommodate multiple passengers, were costly, both in terms of vehicle procurement and maintenance. For example, in the Ugandan RESCUER program, the proportion of supervised births from 1995 to 1998 increased from 15% to 27%, and hospital-based maternal case fatalities were reduced by 50% [46]. However, by 2005, when the program was scaled up to 56 districts, the high cost of vehicle maintenance made sustaining the program difficult because of insufficient funds. The one included study that reported on cost found that using motorcycles was a budget-friendly option. In Ghana, motorcycle ambulances were effective, culturally acceptable, and able to navigate small roads in rough terrain, allowing drivers to reach women in locations otherwise inaccessible by larger vehicles [38]. However, in Malawi, cultural beliefs that publicizing labor could summon evil spirits resulted in infrequent use of bicycle ambulances [41]. For both motorcycle and bicycle ambulances, customized vehicle features like an enclosed carriage/privacy screen, mattress, seat belts, and/or extended mirrors offered improved safety and comfort. These findings suggest that transportation interventions must be adapted according to local physical, social, cultural and economic environments.

Evidence from the included studies suggest that emergency obstetric transportation interventions were more effective when integrated within an enhanced referral system or when additional strategic interventions aimed at improving the quality of care at service delivery points are present. This underscores the need for a pragmatic approach to strengthening the healthcare systems at facility and community points while addressing context specific emergency transportation barriers [47,48]. For example, the intervention described in De Costa et al. [37] included financial support for referrals, incentives for early registration of pregnancy, and training of paramedical staff and traditional birth attendants (TBAs). Similarly, in Uganda, transportation interventions included free-of-charge ambulance service and the provision of 2-way radios, landlines, or cell phones between health facilities [44]. Patel et al. [38] attributed reduced maternal mortality and increased referrals to the use of a 3-wheeled motorcycle and dual-SIM mobile phones. However, no significant effect was observed on the number of facility deliveries and the caesarian delivery rate due to low rates of adherence to some care protocols. While transportation and communication were addressed, the remaining gap in delivery of quality care may have hampered the overall outcomes [38]. The conceptual framework we adopted for this review highlights how underpinning factors of socioeconomic status, accessibility of facilities, and quality of care correlate with each phase of the three-delay model [11].

### Limitations and Implications for Practice and Future Research

The strength of the evidence in this review is limited by the paucity of high-quality eligible studies. There was only one high-quality cluster randomized study, which demonstrated that emergency obstetric transportation intervention could increase caesarean sections and facility delivery. Three of the nine included studies were assessed as having low risk of bias on all domains. We found that most interventions occurred in rural communities, and though women in urban settings have higher odds of facility-based delivery, it is important to note that disparities in access to emergency obstetric care also exist among the urban poor [49]. This underscores the need for inclusion of the urban poor in interventions that seek to increase access to emergency obstetric transportation to reduce adverse pregnancy outcomes. Six of the included studies were conducted in Africa, limiting generalizability of the findings. Finally, although there is promise for transport interventions to reduce transport time and increase receipt of obstetric care, the cost-effectiveness and sustainability of such interventions must be assessed before recommendations for wide-scale implementation can be made. Indeed, only three studies reported running costs of transportation, which may be substantial and often prohibitive in low-resource settings [41,42,44].

Accessing newly established transportation services was an issue reported by several of the studies related to the transportation type as well as other factors like driver motivation. Patel et al. reported that health workers perceived motorcycle ambulance drivers as “very dedicated” (56%) or “somewhat dedicated” (41%), but they also wanted to be trained to operate the motorcycle themselves, so the vehicle could be used if the driver was unreachable [38]. Driver availability was especially challenging at night, with increased delays due to driver mobility and reluctance to travel at night [42]. Some drivers feared for their safety at night, especially motorcycle drivers, who are exposed [38]. Some solutions included providing a special night-out allowance, arranging transportation for the driver to and from their home, and creating a duty schedule and on-call sleeping room at the health facility [42,45]. Other nighttime delays were difficulties obtaining consent from relatives and lack of health workers at the facility [41,44]. Hofman et al. [42] reported that an ambulance was usually available for use within 15 minutes of decision to refer during the day, but much longer for pregnant women presenting between 5 p.m. and 6 a.m. [42] Driver availability and night-specific challenges should be addressed in future transportation interventions.

Equitable access to transportation or transportation funds was also an issue. Certain women may benefit from a particular transportation intervention based on their socioeconomic status or rurality. Decisions about where to station ambulances sometimes contributed to disparities in use between communities. In Uganda, one 4 × 4 ambulance was stationed at the district hospital (referral facility), which admittedly was not centrally located in the district, meaning that the to-and-fro time from the referral facility to the peripheral facilities where emergency obstetric cases originated, ranged from 30 minutes to three hours [44]. The vehicle could only retrieve women from health facilities, not their homes [43,44]. Fournier et al. reported that district accessibility was classified as “good,” “average,” or “poor” based on the proportion of residents (>85%, 60–85%, <60%) that lived within 15 kilometers of a primary health care center [43]. In the intervention year, 39.2% of women with obstetric emergencies came from areas with poor accessibility compared with 17.1% the year prior, meaning the intervention benefitted women from more rural areas [43]. Women who live closer to health care facilities may feel like they have more transportation options if they perceive the waiting time for the ambulance to be long, whereas women from rural areas may have fewer options. De Costa et al. reported low uptake of transportation funds (23.8% of high-risk women who received a referral availed the cash for transportation benefit) and attributed this to social hierarchies in the community; the funds were distributed by a gatekeeper, whom they described as “socially better placed” than many of the intervention participants, which they speculated limited the interaction between the groups [37]. In Uganda, transportation vouchers were in high-demand, so they were not always available when needed. Additionally, the village health teams who issued the vouchers did not always adhere to eligibility criteria, and some of the motorcycle ambulance drivers procured vouchers for resale at higher prices, often resulting in inequitable distribution of vouchers [45].

The vast majority of excluded studies addressed the issue of emergency obstetric transportation but only reported data from interviews with community members about their perceived barriers to care and/or reasons for high mortality rates [50,51,52]. Several studies also measured the success of transport schemes by indicators such as number of people transported to health facilities, number of drivers trained, or community members’ qualitative acceptance of or satisfaction with the transport scheme, which do not necessarily correlate with the overarching goal of transport schemes – reducing adverse health outcomes for mothers and the newborn [53,54]. While increasing the number of women presenting at an appropriate care facility for emergency services is an accomplishment, they may not receive services due to lack of a physician, inability to pay, or medications being unavailable [55]. No studies in this review measured met/unmet EmONC needs. Finally, the most salient factor determining the availability of studies reporting the effectiveness of transport interventions in lowering mortality rates is the difficulty of obtaining reliable mortality data from these remote communities. In isolated, rural communities, where health care utilization is low, adequate recordkeeping of birth, morbidity, and mortality rates may not exist [56].

## Conclusion

Considering the scarcity of evaluative studies on emergency transport interventions, this review highlights the need for future studies to employ a variety of study designs (experimental or quasi-experimental designs of large, multi-sited programs) to assess the impact of transport interventions on mortality rates and other related secondary outcomes. Additional operational research is needed to determine the sustainability and cost-effectiveness of emergency transport interventions before recommending wide-scale implementation. Poor quality of healthcare services and financial constraints hinder optimal use of maternal and newborn health services in many LMICs [57,58]. It is important to examine the relationship between transportation, health care utilization after transportation, and mortality rates. Several challenges have been identified, including high vehicle and maintenance costs, establishing effective communication systems in remote settings, maintaining driver coverage, ensuring equitable access to transportation, and sustainability within a resource-constrained health system. New technologies, such as alternative transportation vehicles or mobile phones, are becoming available in low-income settings and should be evaluated in the context of maternal-newborn health transport systems. Finally, studies need to consistently record process indicators to track program effectiveness such as time to reach referral hospital, which has been found to be critical in reducing maternal and neonatal mortality [59,60].

## Additional File

The additional file for this article can be found as follows:

10.5334/aogh.2934.s1 PRISMA Checklist. Emergency transportation interventions for reducing adverse pregnancy outcomes in LMICs.
